# Identification and Validation of Reliable Reference Genes for Gene Expression Studies in Postharvest Atemoya Pulp (*Annona cherimola* Mill × *A. squamosa* L.) Subjected to Different Preservation Treatments

**DOI:** 10.3390/ijms27188114

**Published:** 2026-09-11

**Authors:** Xueyu Zhang, Qinyi Gao, Ying Zhou, Hanzhou Zhang, Zhihui Chen, Jingjing Chen

**Affiliations:** 1 Key Laboratory of Tropical Fruit Biology, Ministry of Agriculture and Rural Affairs, South Subtropical Crops Research Institute, Chinese Academy of Tropical Agricultural Sciences, Zhanjiang 524091, China; zhangxueyu@catas.cn (X.Z.); gaoqinyi71@163.com (Q.G.); zhouying200211@163.com (Y.Z.); zhhanzhou@163.com (H.Z.); chenzhihui@catas.cn (Z.C.); 2 State Key Laboratory of Tropical Crop Breeding, South Subtropical Crops Research Institute, Chinese Academy of Tropical Agricultural Sciences, Sanya 572024, China

**Keywords:** *Atemoya*, reference genes, postharvest storage

## Abstract

Quantitative real-time PCR (qRT-PCR) is a widely used technique for quantifying gene expression. However, accurate data normalization requires stable reference genes that exhibit constant expression levels across different experimental conditions. To date, no suitable reference genes have been validated for atemoya fruits (*Annona cherimola* Mill × *A. squamosa* L.). This study aimed to evaluate the expression stability of candidate reference genes in atemoya across distinct tissues, postharvest storage stages, and various treatment conditions. To this end, we designed specific primers for six reference genes: *ubiquitin carrier-like protein* (*UBC*), *actin 7* (*ACT7*), *actin 11* (*ACT11*), *elongation factor 1α* (*EF1α*), *18S ribosomal RNA* (*18S*), and *β-tubulin* (*TUB*). Total RNA was extracted from atemoya tissues (e.g., petals, young fruits, and roots), for pulp across three post-harvest time points (0, 2, and 4 days) and under different conditions: 28 °C, 15 °C, ethylene, or 1-MCP treatment. The geNorm tool was subsequently used to identify the most stable reference transcripts under each experimental condition. *ACT7* was the most stable reference gene across atemoya tissues, while *UBC* ranked first under all postharvest conditions tested (28 °C storage, 15 °C cold storage, ethylene, and 1-MCP). Furthermore, the suitability of these reference genes under ethylene and 1-MCP treatments was validated by examining the expression patterns of *AaPG* and *AaERF*. Collectively, the combined use of *ACT7*, *UBC*, and *18S* proved to be the most reliable normalization strategy across all experimental conditions. This validated multi-gene approach provides a robust foundation for future investigations into gene expression and the postharvest ripening mechanisms of atemoya.

## 1. Introduction

Atemoya is a fruit tree belonging to the Annonaceae family. It is an interspecific hybrid derived from a cross between the cherimoya (*Annona cherimola* Mill.) and the sugar apple (*A. squamosa* L.), also known locally as “pinha” or “fruta-do-conde”. The initial hybridization was performed in Florida (USA) in the early 20th century, and subsequent crosses were carried out in other countries to develop cultivars adapted to different climatic conditions—tropical for *A. squamosa*-like traits and subtropical for *A. cherimola*-like traits [1]. Atemoya is a climacteric fruit characterized by high respiration rates and elevated ethylene production, which collectively accelerate postharvest deterioration. This rapid quality loss severely limits its commercial potential, underscoring the urgent need for effective technologies to delay fruit ripening [2]. Appropriate low-temperature storage or treatment with ethylene inhibitors such as 1-MCP can effectively delay fruit ripening [3,4]. Understanding the molecular mechanisms underlying these postharvest processes requires transcriptional profiling of genes differentially expressed during storage. Previous studies on cherimoya, sugar apple, and atemoya have employed *actin* as a reference gene for gene expression analyses [2,5,6], and the stability of reference genes in soursop (*Annona muricata* L.) has also been evaluated [7]. However, to date, no systematic validation of reference genes has been reported for atemoya fruits. Therefore, the identification and validation of stably expressed internal controls in atemoya is essential for accurate gene expression normalization in future postharvest studies.

Quantitative real-time PCR (qRT-PCR) is the most widely used technique for measuring gene expression patterns and validating RNA-seq data. However, accurate normalization is required to correct for variations in target gene mRNA levels by using a suitable reference gene. Since no universal reference gene exists, the reliability of normalization ultimately depends on the reference genes chosen for each experimental context. Hence, reference gene selection and validation must be performed for each organism and experimental condition, as endogenous gene expression varies across species and treatments. Therefore, the expression stability of candidate reference genes should be rigorously validated to identify the most suitable internal control [8]. A reference gene is defined as a gene that maintains stable expression at a constant level across diverse experimental conditions, tissues, organs, and other biological contexts, and is expected to remain unaffected by the treatments under investigation. The most commonly used reference genes for data normalization in plants include *ubiquitin carrier-like protein* (*UBC*), *β-actin* (*actin*), *elongation factor 1α* (*EF1α*), and *18S ribosomal RNA* (*18S*), among others. However, the expression levels of reference genes can vary significantly from one experimental condition to another. Therefore, it is essential to select and validate stably expressed internal reference genes for accurate gene expression analysis in atemoya fruits under different storage conditions. This study was conducted to identify suitable housekeeping genes for normalizing gene expression in atemoya fruits subjected to different postharvest treatments. We evaluated the expression stability of six candidate reference genes (*UBC*, *ACT7*, *ACT11*, *EF1α*, *18S*, and *TUB*) in atemoya flesh under various experimental conditions using the geNorm algorithm implemented in R (version 4.6.1) [9]. The present study was designed to identify robust reference genes that maintain stable expression across a range of experimental conditions, including different tissues, fruits of varying maturity, and fruits undergoing distinct postharvest manipulations. Ultimately, this work aims to support more accurate and reliable gene expression analyses in the postharvest biology of atemoya, thereby contributing to better understanding of its quality maintenance and shelf-life extension.

## 2. Results

### 2.1. Selection of Candidate Reference Genes and Amplification Specificity RNA

**Figure 1:**
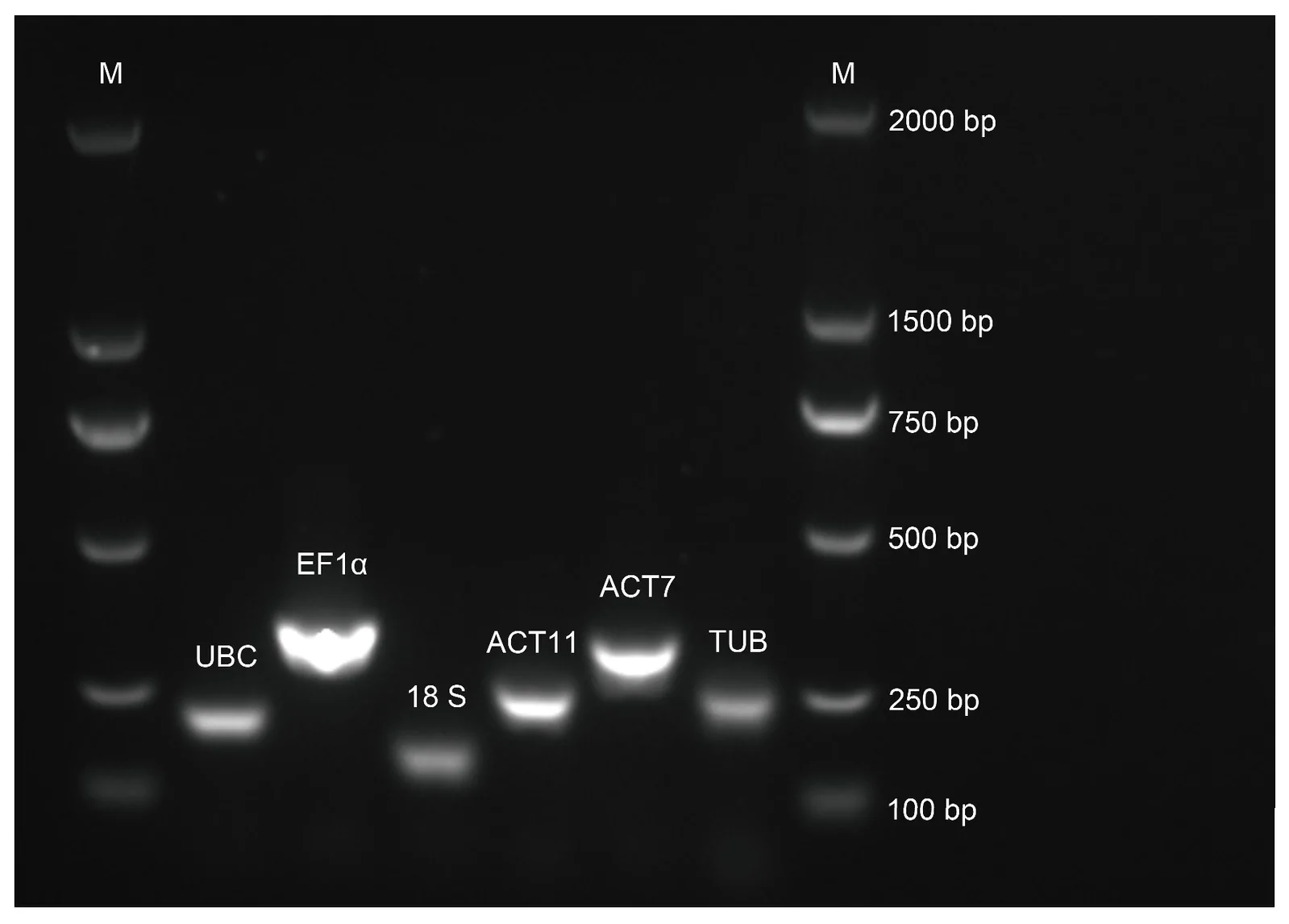
Electrophoretic analysis of the amplified fragments of six candidate reference genes (*ACT7*, *ACT11*, *UBC*, *18S*, *EF1α*, and *TUB*) on a 1% agarose gel. M indicates the DL2000 DNA marker.

**Figure 2:**
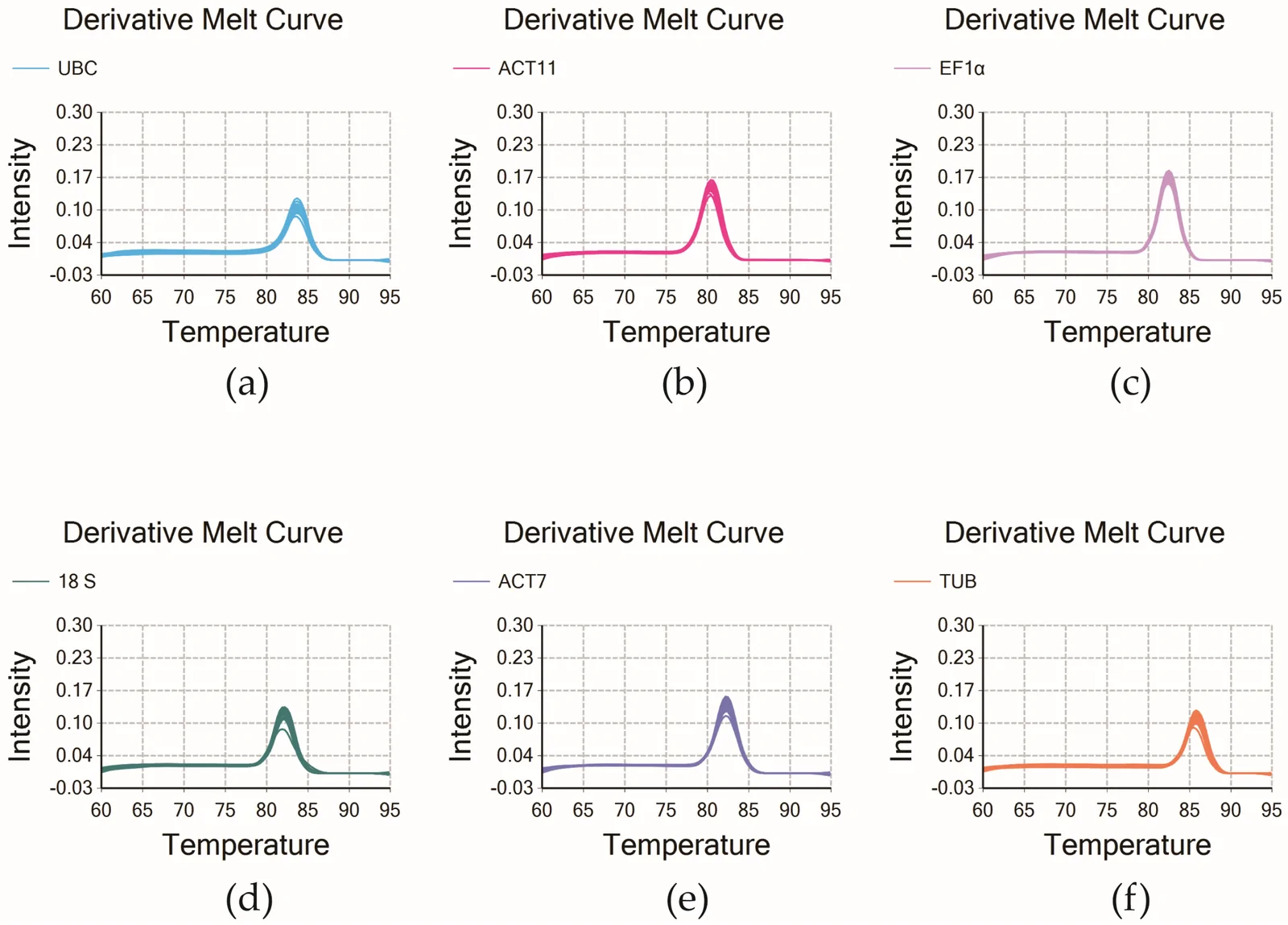
Melting curve analysis of the six candidate reference genes (**a**) *UBC*; (**b**) *ACT11*; (**c**) *EF1α*; (**d**) *18S*; (**e**) *ACT7*; (**f**) *TUB* as determined by qRT-PCR. Each curve represents a single peak, indicating specific amplification without primer-dimer formation or non-specific products.

Total RNA was isolated from samples across all experimental conditions, and first-strand cDNA was generated via reverse transcription. The amplification of six candidate reference genes (*UBC*, *ACT7*, *ACT11*, *EF1α*, *TUB*, and *18S*) was performed on this cDNA using conventional PCR with sequence-specific primers, which successfully produced distinct amplicons for all primer pairs. Electrophoretic analysis revealed a distinct single band for each PCR product, with mobilities corresponding to the anticipated amplicon sizes, thereby validating the specificity of all primer sets (Figure 1). The six candidate genes were subjected to qRT-PCR analysis using the six primer pairs, which were found to have amplification efficiencies ranging from 90.61% to 106.68%, and the standard curve correlation coefficients (R^2^) ranged from 0.98 to 0.99 (Table 1; Supplementary Table S1). The specificity of the qRT-PCR primers was corroborated through both melting curve analysis and agarose gel electrophoresis. Each amplicon exhibited a homogeneous melting profile with a single peak, and corresponding electrophoretic profiles showed discrete bands devoid of primer-dimer species, collectively attesting to the high specificity of the assays (Figure 2). Having been validated, these primer sets were deemed suitable for subsequent quantitative real-time PCR (qRT-PCR) applications.

### 2.2. Expression Levels of Candidate Reference Genes

**Figure 3:**
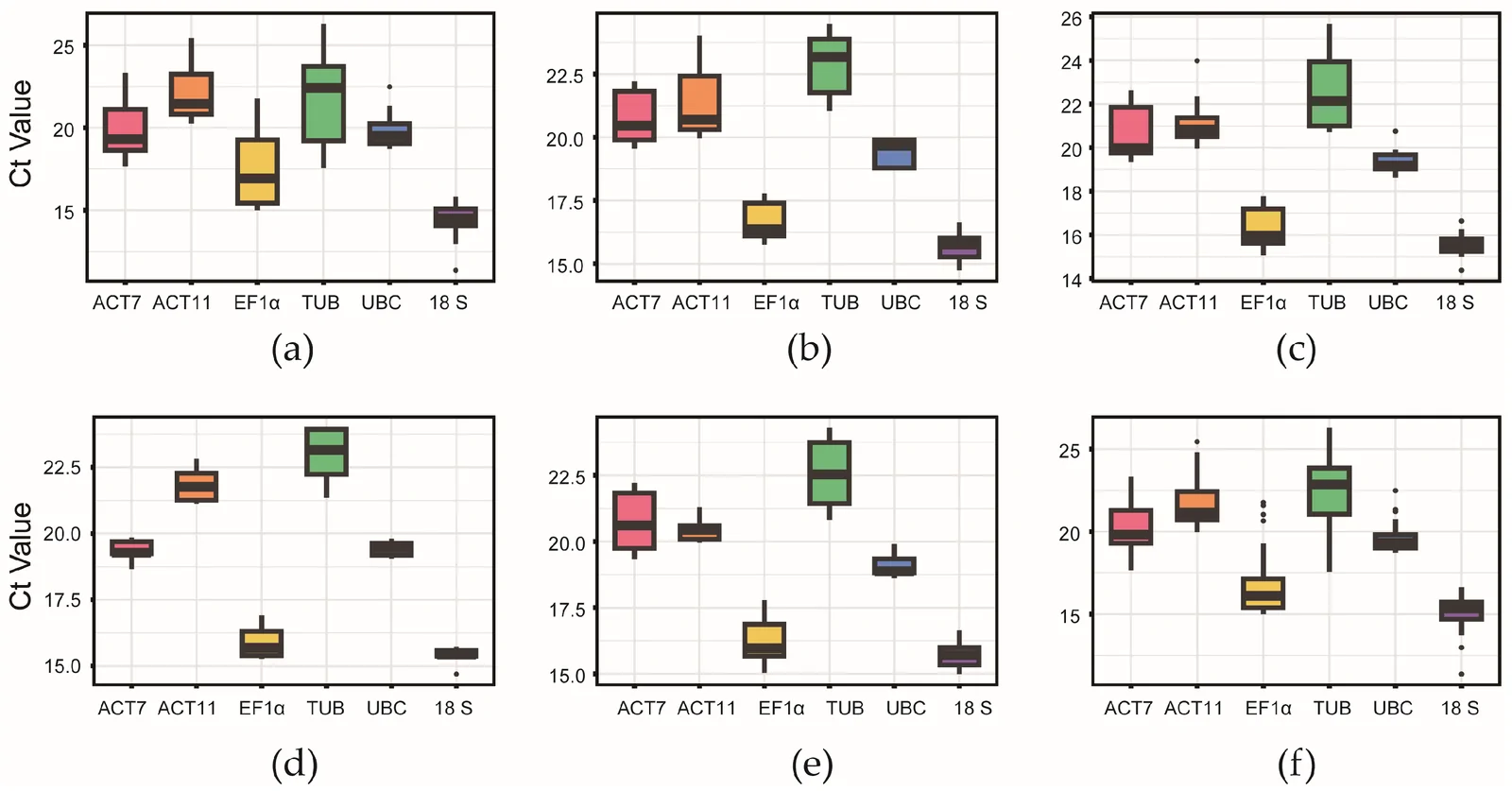
Box plots showing the distribution of Ct values for the candidate reference genes across different sample sets: (**a**) various tissues, (**b**) different fruit maturation stages, (**c**) fruit subjected to low-temperature treatment, (**d**) ethylene-treated fruit, (**e**) 1-MCP-treated fruit, and (**f**) all sample. The boxes represent the interquartile range (25th to 75th percentile), whiskers indicate the minimum and maximum Ct values, and the horizontal line within each box denotes the median.

The threshold cycle (Ct) values were analyzed using qRT-PCR in all conditions. The mean Ct values of the reference genes varied across different experimental sets: from 14.39 (*18S*) to 22.16 (*ACT11*) in different tissues; from 15.61 (*18S*) to 22.98 (*TUB*) in fruits at different maturity stages during postharvest storage; from 15.56 (*18S*) to 22.55 (*TUB*) in cold-stored fruits; from 15.38 (*18S*) to 22.96 (*TUB*) in ethylene-treated fruits; from 15.71 (*18S*) to 22.53 (*TUB*) in 1-MCP-treated fruits; and from 15.09 (*18S*) to 22.35 (*TUB*) across all conditions combined (Figure 3). Notably, when all samples from both untreated and treated fruits were pooled, *18S* showed the highest transcript abundance (lowest Ct, 15.09), while *TUB* exhibited the lowest expression level (highest Ct, 22.35) (Supplementary Tables S2–S6).

### 2.3. Transcript Stability of Candidate Reference Genes Ct

To determine the most suitable reference genes for normalizing gene expression under our experimental conditions, we assessed the stability of candidate genes based on their Ct values. The stability rankings were generated using both the geNorm algorithm [10] and the RefFinder online tool [11], with the results summarized in Table 2 and Supplementary Tables S2–S6. From the geNorm analysis, we calculated the M value to identify the most stably expressed transcripts, and the V value to determine the optimal number of reference genes. Following the criterion proposed by Vandesompele et al. (2002) [12], we applied a cut-off of 0.15 for Vn/n + 1, meaning that any value below this threshold indicates that adding more reference genes would not yield a significant improvement in normalization.

#### 2.3.1. Different Tissue Types in Atemoya

**Figure 4:**
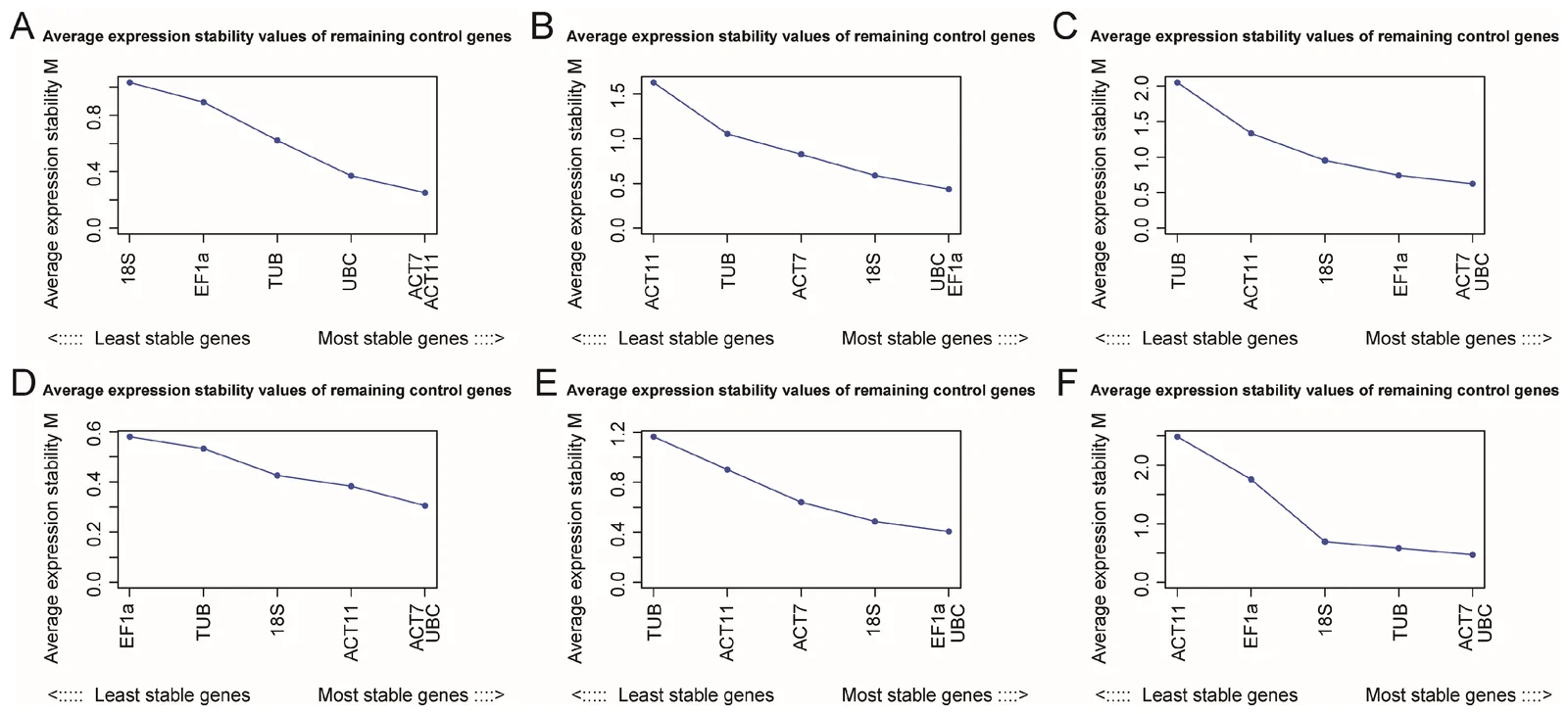
Average expression stability (M) calculated by geNorm. A lower M-value indicates more stable gene expression: (**A**) various tissues (petals, young fruits, mature fruits, branches, roots, and leaves), (**B**) different fruit maturation stages, (**C**) fruit subjected to low-temperature treatment, (**D**) ethylene-treated fruit, (**E**) 1-MCP-treated fruit, and (**F**) all sample.

**Figure 5:**
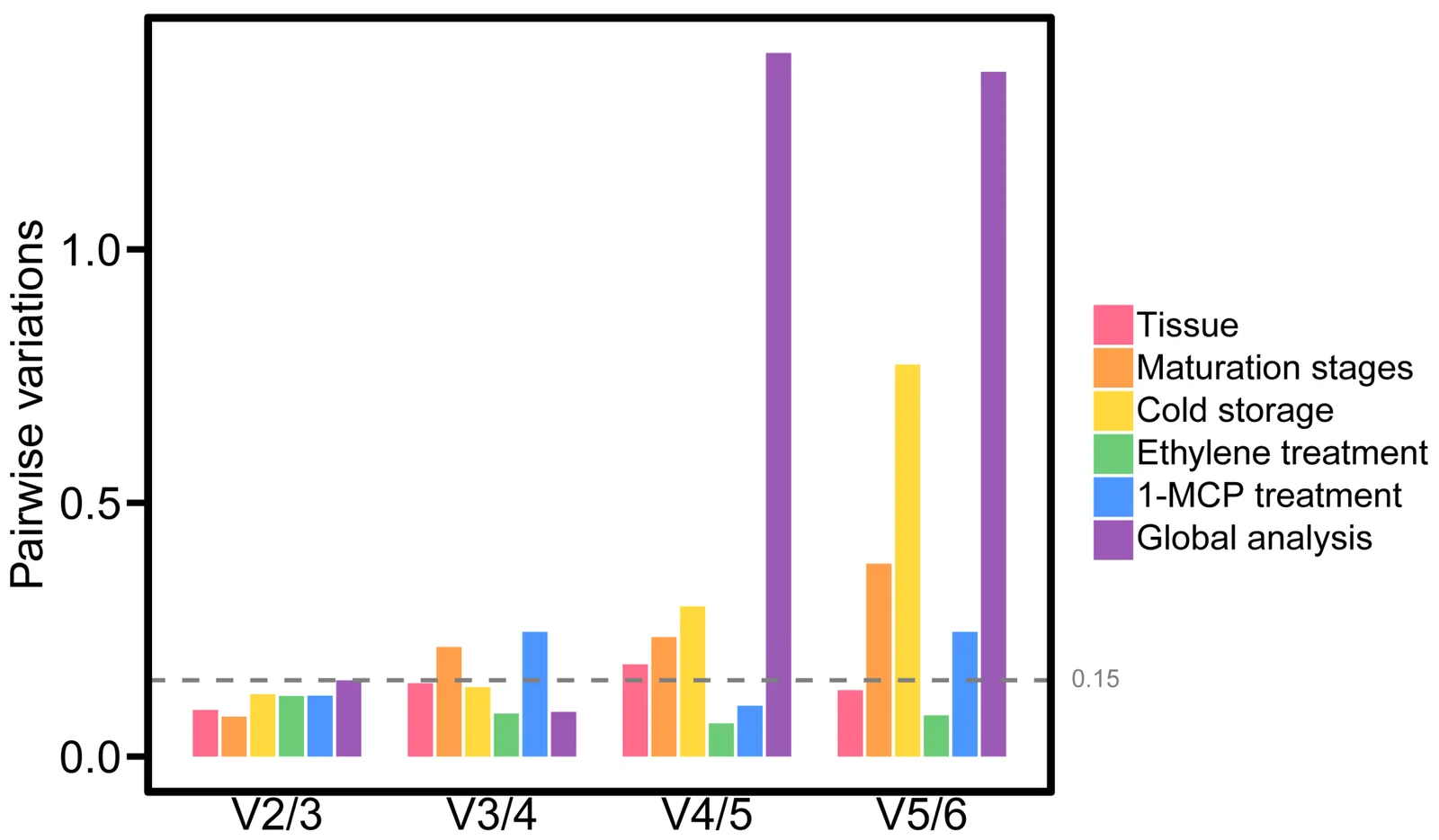
Determination of the optimal number of reference genes by pairwise variation analysis using geNorm. Pairwise variation (Vn/Vn + 1) was analyzed between the normalization factors NFn and NFn + 1, in all conditions tested. Tissues: petals, young fruits, mature fruits, branches, roots, and leaves.

According to the geNorm analysis, *ACT11*/*ACT7* exhibited the highest transcript stability across different atemoya tissues, whereas *18S* was the least stable (Figure 4). This result was largely supported by both the ΔCt method and Normfinder, which consistently identified *ACT11*, *ACT7* and *UBC* as the most stable transcript. In contrast, BestKeeper produced a different ranking, placing *18S* first, followed by *TUB*. The comprehensive stability ranking generated by RefFinder, integrating all four algorithms, was as follows (from most to least stable): *ACT11* > *ACT7* > *UBC* > *TUB* > *18S* > *EF1α* (Table 2). As shown in Figure 5, the geNorm pairwise variation (V) analysis yielded a V2/3 value of 0.0916, which is below the 0.15 threshold, confirming that two reference genes (*ACT11* and *ACT7*) are sufficient for accurate normalization of qRT-PCR data across different atemoya tissue types examined.

#### 2.3.2. Different Ripening Stages of Atemoya Fruit

When evaluating reference gene performance during fruit ripening, geNorm identified *UBC*, *EF1α* and *18S* as the most stable pair, whereas *ACT11* and *TUB* was the least reliable (Figure 4). This pattern was largely supported by ΔCt and NormFinder methods, which consistently ranked *EF1α* as the most stable transcript and *ACT11* as the least. BestKeeper analysis indicated *18S* as the most stable and *UBC* as the second most stable. A comprehensive ranking based on RefFinder yielded an overall consensus order for ripening stages: *UBC* > *EF1α* > *18S* > *ACT7* > *TUB* > *ACT11* (Table 2). The pairwise variation coefficient V2/3 (0.0783) calculated by geNorm fell below the accepted threshold of 0.15 (Figure 5), indicating that normalization of qRT-PCR data across ripening stages requires using *UBC* and *EF1α* together.

#### 2.3.3. Low-Temperature-Treated Atemoya Fruit

Under cold storage conditions (15 °C), geNorm ranked *UBC* and *ACT7* as the most stable transcripts (Figure 4). NormFinder and ΔCt methods also identified *UBC*/*ACT7* as the most stable transcript, with *TUB* being the least stable (Table 2). However, BestKeeper analysis indicated *18S* as the most stable and *UBC* as the second most stable. Consistent with the other algorithms, RefFinder results showed *UBC* to be the most stable transcript, yielding the following ranking (from most to least stable) in cold-stored fruits: *UBC* > *18S* > *EF1α* > *ACT7* > *ACT11* > *TUB* (Table 2). The pairwise variation coefficient V2/3 (0.1223) under cold storage was below the recommended cutoff of 0.15 (Figure 5), indicating that two reference genes (*UBC* and *18S*) are required for reliable normalization of gene expression levels in cold storage fruits.

#### 2.3.4. Ethylene-Treated Atemoya Fruit

Following ethylene treatment, geNorm analysis identified *UBC* and *ACT7* as the most stable reference genes, whereas *EF1α* and *TUB* exhibited the lowest stability (Figure 4). This result was largely supported by both the ΔCt method and Normfinder, which respectively ranked *UBC* and *ACT7* as the top reliable transcripts. However, BestKeeper analysis indicated *18S* as the most stable and *UBC* as the second most stable. The comprehensive ranking derived from RefFinder, integrating all four algorithms, placed the candidate genes in the following order for ethylene-treated fruit: *UBC* > *18S* > *ACT7* > *TUB* > *ACT11* > *EF1α* (Table 2). Moreover, the geNorm pairwise variation analysis (Figure 5) revealed that the V2/3 values (0.1188) fell below the recommended cutoff of 0.15, indicating that the two-gene combination of *UBC* and *18S* is sufficient for reliable normalization of qRT-PCR data under ethylene treatment conditions.

#### 2.3.5. 1-MCP-Treated Atemoya Fruit

In the case of 1-MCP-treated fruit, geNorm and the ΔCt approach ranked *UBC* and *EF1α* as the most stable transcripts, with *TUB* falling at the bottom of the stability list (Figure 4). The NormFinder analysis indicated *EF1α* as the most stable and *UBC* as the second most stable candidate. BestKeeper diverged from the other algorithms by placing *ACT11* at the top and *18S* in second place. The integrated RefFinder analysis produced a final stability ranking for 1-MCP-treated samples as follows: *UBC* > *18S* > *EF1α* > *ACT11* > *ACT7* > *TUB* (Table 2). Additionally, the geNorm pairwise variation analysis (Figure 5) showed that the V2/3 ratios (0.1196) fell below the 0.15 cutoff, thereby emphasizing the requirement to use *UBC* and *18S* as a two-gene reference set for reliable data normalization under 1-MCP treatment conditions.

#### 2.3.6. Global Analysis

For the entire dataset, the geNorm method yielded M values ranging from 1.85 to 3.95, with *UBC* and *ACT7* being the most stable transcripts and *ACT11* the least stable (Figure 4). The ΔCt method and NormFinder consistently ranked *UBC* as the most stable transcript, while *ACT11* were identified as the least stable. However, BestKeeper showed *18S* as the most stable and ACT7 as the second most stable candidate. Across all conditions, the overall stability ranking generated by RefFinder (from most to least stable) was: *ACT7* > *UBC* > *18S* > *TUB* > *EF1α* > *ACT11* (Table 2). The global analysis also showed V2/3 values (0.1496) below the 0.15 cutoff (Figure 5), indicating that the two reference genes (*ACT7* and *UBC*) should be used together for reliable normalization of gene expression under all conditions tested.

#### 2.3.7. Robustness Evaluation of Relative Expression Across Different Internal Controls

**Figure 6:**
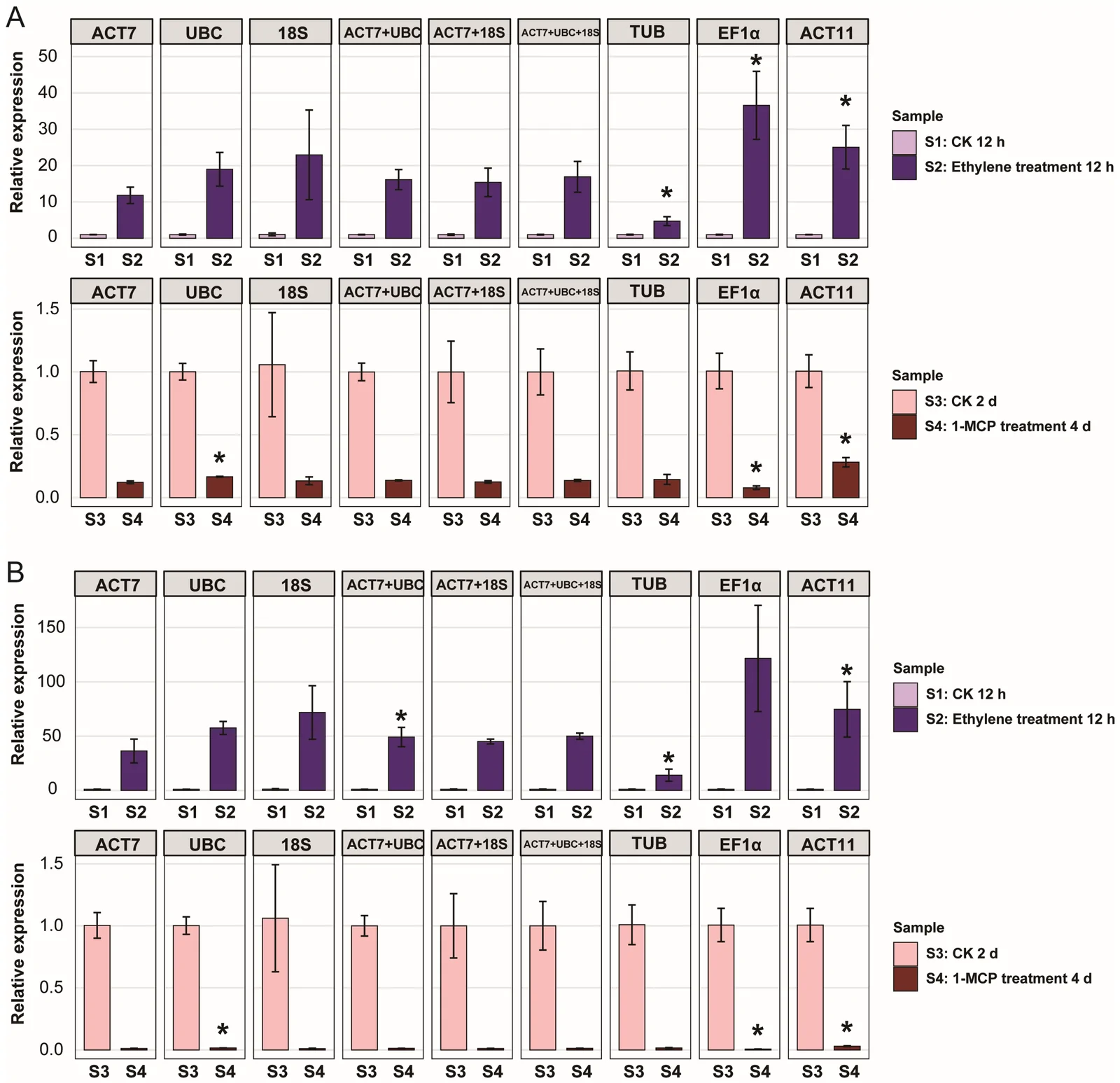
Relative expression of *AaPG* and *AaERF* in fruits at different treatment with *A. atemoya*. (**A**) *AaPG*, (**B**) *AaERF*. Asterisks (*) indicate significant differences (*p* < 0.05) relative to the value normalized with ACT7 at each sample. CK: untreated control.

The expression levels of *PG* (polygalacturonase) and the transcription factor *ERF* (ethylene responsive factor) in climacteric fruits in response to ethylene and the ethylene inhibitor 1-MCP were calculated using the reference genes recommended above. Based on the evaluation results, *ACT7* was identified as the most stable reference gene, while *ACT11* was the least stable among the six candidate genes. *UBC* and *18S* were also favored by two of the three algorithms, although *18S* exhibited relatively lower stability compared with *ACT7* and *UBC*. As shown in Figure 6 and Supplementary Tables S7 and S8, when normalized to *ACT7*, the transcript level of *AaPG* was up-regulated by 11.8-fold at 12 h after ethylene treatment relative to untreated controls, and down-regulated by 8.2-fold at 4 days after 1-MCP treatment relative to untreated controls. Similar expression trends were observed when normalization was performed using 18S alone or in combination with *ACT7*. In contrast, when *EF1α* or *ACT11* was used as the reference, the ethylene-induced up-regulation of *AaPG* was estimated at 36.5-fold and 25.0-fold, respectively, both of which were significantly different from the value obtained with *ACT7* (Figure 6). Normalization with *TUB* yielded an ethylene-induced up-regulation of only 4.7-fold, also significantly different from the *ACT7*-normalized value (Figure 6). For 1-MCP treatment, normalization with *EF1α* or *ACT11* resulted in down-regulation values of 12.8-fold and 3.6-fold, respectively, both significantly different from the *ACT7*-normalized result (Figure 6). Notably, normalization with UBC alone also produced a significant difference, despite a down-regulation of 6.0-fold. In contrast, normalization with *ACT7*, *18S*, *ACT7* + *UBC*, *ACT7* + *18S*, or *ACT7* + *UBC* + *18S* yielded no statistically significant differences. The *ERF* transcription factor exhibited more pronounced changes in ethylene or 1-MCP-treated fruit. When normalized to *ACT7*, its transcript level was up-regulated by 36.1-fold at 12 h after ethylene treatment relative to untreated controls, and down-regulated by 82.4-fold at 4 days after 1-MCP treatment. Comparable expression patterns were obtained using *18S* alone or the *ACT7* + *18S* combination. However, normalization with *ACT11* gave an ethylene-induced up-regulation of 74.2-fold, which was significantly different from the *ACT7*-normalized value (Figure 6). Additionally, normalization with *UBC* alone also yielded a significant difference, with a down-regulation of 62.5-fold, whereas no significant differences were observed when using *ACT7* alone, *18S*, *TUB*, or any combination of *ACT7* with other reference genes. Collectively, these findings indicate that normalization using the three most stable reference genes in combination—particularly when including *ACT7*—yielded reliable and reproducible expression patterns for both *AaPG* and *AaERF*, further supporting the suitability of these reference genes for accurate transcriptional analysis in this experimental system.

## 3. Discussion

To ensure the reliability of qRT-PCR-based quantification in plant gene expression studies, proper normalization with suitable reference genes is critical, as the stability of these endogenous controls directly influences the accuracy of the analytical outcomes. Historically, commonly employed housekeeping genes—including *ACTIN*, *TUB*, *EF1α*, and *18S rRNA*—have been routinely utilized for data normalization. Nevertheless, a growing body of evidence indicates that the expression of these conventional reference genes is not constitutively invariant across diverse plant species, tissue types, or abiotic and biotic stress regimes [8]. Consequently, it has become an essential prerequisite to systematically evaluate and validate candidate reference genes under the specific experimental conditions and plant materials under investigation prior to conducting qRT-PCR assays, thereby safeguarding the integrity and reproducibility of the resultant expression data. Atemoya, a typical climacteric tropical fruit, undergoes rapid postharvest quality deterioration, and current storage and transportation technologies remain far from optimal. In recent years, the widespread application of transcriptome sequencing has greatly advanced the understanding of molecular regulatory mechanisms underlying postharvest physiology in atemoya fruit, with qRT-PCR being routinely employed for quantitative validation of gene expression [2,13,14,15]. However, a notable gap persists in this species, as systematic screening and evaluation of reference genes have yet to be conducted. Given that the expression stability of conventional housekeeping genes varies considerably across different species, tissues, and treatment conditions, direct adoption of reference genes reported in other organisms may introduce substantial biases or even erroneous interpretations of target gene quantification. Therefore, prior to performing qRT-PCR analysis under specific postharvest conditions in atemoya fruit, it is imperative to establish a dedicated and rigorously validated reference gene system, which serves as a fundamental prerequisite for ensuring the scientific credibility and reliability of expression data interpretation.

In recent years, an increasing number of studies have reported reference gene validation in fruit tree species, including soursop [7], apple [8], cherry [9], watermelon [16], and pineapple [17]. Screening stably expressed reference genes using algorithms such as geNorm, NormFinder, BestKeeper, and RefFinder has become a common practice in various economically important plants [18]. In the present study, we used atemoya as the experimental material and systematically evaluated the expression stability of six candidate reference genes under a range of conditions, including different tissues, fruits at varying maturity stages, cold-stored fruits, and fruits treated with ethylene or 1-MCP. These findings provide a valuable resource for future gene expression analyses involving tissue-specific and postharvest-related studies in atemoya. In plant molecular studies, qRT-PCR is a widely used tool for profiling gene expression under diverse physiological and environmental conditions, and the selection of appropriate reference genes is critical for obtaining reliable results [19]. Transcriptomic studies on atemoya (fruit ripening [14,15] and cracking [2,13]), cherimoya (postharvest [6]) and sugar apple (flowering [5]) have used *ACT11* as a reference gene, while cherimoya chilling injury research [20,21] has used UBC. However, none of these studies validated the stability of their chosen reference genes, and such validation remains absent for atemoya. Notably, a reference gene validation study in soursop (*Annona muricata* L.)—the species most closely related to atemoya within the genus Annona, albeit a distinct species—identified *UBC* and *EF1α* as the most stable transcripts, which differs from the findings of the present study in atemoya. This discrepancy suggests that reference gene stability data from even closely related species cannot be directly extrapolated to atemoya. Therefore, it is of considerable significance to systematically validate the expression stability of reference genes specifically in atemoya, thereby establishing a solid foundation for accurate gene expression analyses in this economically important fruit species. Beyond the stable reference genes reported in Annona (*UBC*, *ACT11*, *EF1α*), *ACT7* was stable in apple flesh [8], *18S rRNA* in Populus [22], and *TUB* in tomato [23]. We thus identified their atemoya orthologs via homology-based BLAST (TBtools v2.483) and evaluated their stability accordingly. In this study, the amplification efficiencies of the six candidate reference genes ranged from 90.61% to 103.14%, all of which fell within the acceptable range for qRT-PCR analysis. In addition, all melting curves exhibited single peaks, and PCR product electrophoresis on agarose gels yielded single bands for each primer pair, indicating that the designed primers had good amplification specificity. Among the candidate genes, *18S* exhibited a relatively low mean Ct value, suggesting high expression abundance, whereas TUB showed a relatively high mean Ct value, indicating relatively low expression abundance. It should be noted, however, that Ct values merely reflect transcript abundance and do not directly denote expression stability; the latter must be comprehensively evaluated based on the expression fluctuation patterns of each gene across different tissues and treatments. In the present study, the Ct values of the six candidate reference genes were obtained under various experimental conditions, from which the Q-values were subsequently calculated. Based on these Q-values, the stability of each gene was assessed using multiple algorithms, and the rankings generated by different algorithms were comprehensively evaluated to obtain a final consensus ranking, which identified *ACT7*, *UBC*, and *18S* as the most stable reference genes across all conditions tested. This consensus ranking was further subjected to validation analysis. Notably, the rankings obtained under different experimental conditions remained largely consistent across most of the algorithms employed, reinforcing the robustness of our findings. However, a detailed examination of the rankings across specific experimental subsets revealed that no single gene exhibited optimal stability under all conditions. For instance, *ACT7* and *ACT11* emerged as reliable reference genes for normalizing gene expression across different tissues, yet *ACT11* showed considerably poor stability in fruits subjected to different postharvest treatments. Conversely, *UBC* was found to be more suitable for postharvest-related studies, but its stability was remarkably poor across different tissue types. These observations indicated that a single reference gene was insufficient to account for the complex transcriptional variations across different fruit tissues and storage stages. Collectively, the recommended reference gene panels established in this study—which advocate for the combined use of three reference genes, *ACT7*, *UBC*, and *18S*—provide a solid foundation for future investigations into the molecular mechanisms underlying tissue development, fruit ripening, and postharvest physiology in atemoya.

Despite the systematic evaluation of six candidate reference genes for their expression stability across different atemoya tissues and postharvest treatment conditions, and the identification of reliable reference gene combinations, several limitations of this study should be acknowledged. First, the number of candidate reference genes examined was limited to six; although these genes represent commonly used housekeeping genes in plant studies (e.g., *Actin*, *TUB*, *EF1α*, and 18S rRNA), the relatively small panel size may not fully capture the most stable transcripts present in the atemoya transcriptome, and future studies could mine a wider range of potential reference genes from large-scale transcriptomic datasets to further enhance normalization robustness. Second, while we have supplemented our study with functional validation experiments using two target genes, *AaPG* and *AaERF* (see Section 2.3.7 and Figure 6), which confirmed the effectiveness of the recommended reference genes, we acknowledge that other key ripening-related genes such as *ACS* and *ACO* remain to be tested in future work. Third, our experiments were conducted using a single cultivar and under specific storage temperatures and chemical treatments; the stability rankings may differ under other cultivars, temperature regimes, or alternative postharvest interventions (e.g., modified atmosphere storage, coating preservation, or UV irradiation), so caution should be exercised when generalizing our conclusions to broader postharvest scenarios. In summary, while this study provides valuable guidance for reference gene selection in postharvest research on atemoya, these limitations highlight the importance of conducting pilot experiments under specific conditions and adopting a multi-reference-gene normalization strategy to ensure accurate and biologically meaningful gene expression quantification.

## 4. Materials and Methods

### 4.1. Plant Material

After harvest, atemoya fruits were subjected to different postharvest treatments. Fruits stored at 28 °C in an artificial climate chamber (DRX-280D-LED, KESHENG, Ningbo, China) were sampled at 0 day, 12 h, 2 days, and 4 days post-storage (different fruit maturation stages). Fruits stored at 15 °C in an artificial climate chamber were sampled at 2 days and 4 days post-storage (low-temperature treatment). For ethylene treatment, fruits were dipped in 1 g/kg ethephon solution (B62581, Yuanye Bio-Technology Co., Ltd., Shanghai, China) for 10 min and sampled at 12 h post-treatment. For 1-MCP treatment, fruits were exposed to 1.8 µL/L 1-MCP (S25973, Yuanye Bio-Technology Co., Ltd., Shanghai, China) in a sealed chamber for 24 h at room temperature, after which the chamber was ventilated, and samples were collected at 2 days and 4 days post-treatment. At each time point, pulp tissues from three fruits were pooled to generate one biological replicate, and three such replicates were independently prepared, snap-frozen in liquid nitrogen, and stored at −80 °C until further use. Different tissues of atemoya, namely petals, young fruits, mature fruits, branches, roots, and leaves, were harvested from healthy trees grown at the South Subtropical Crops Research Institute (Zhanjiang, Guangdong, China). All collected samples were promptly snap-frozen in liquid nitrogen and subsequently stored at −80 °C until RNA extraction.

### 4.2. RNA Extraction and First cDNA Synthesis

Total RNA was extracted from various plant organs (i.e., roots, branches, leaves, petals, young fruits, and mature fruits), and the resulting samples were used as tissue materials for expression stability analysis across different plant parts. Total RNA was isolated from atemoya tissues using the Polymer Rich Plant RNA Extraction Kit (CT8063A, Cinotohi, Changsha, China) following the manufacturer’s instructions. The quality and integrity of the extracted RNA were assessed by 1% agarose gel electrophoresis and spectrophotometric analysis (NP3000, LabYeah, Shanghai, China), with A260/A280 and A260/A230 ratios ranging from 1.8 to 2.1 indicating acceptable purity. For cDNA synthesis, 1 µg of total RNA was reverse-transcribed using the TransScript One-Step gDNA Removal and cDNA Synthesis SuperMix (AH311; TransGen Biotech, Beijing, China) according to the manufacturer’s protocol. The synthesized cDNA was stored at −20 °C until further use.

### 4.3. Selection of Reference Genes and Primer Design

Bioinformatics analysis was performed to identify putative reference genes in atemoya. Given the phylogenetic proximity and the availability of genomic resources, we referred to reference genes previously used in soursop, apple, cherry, and other plant species. Candidate gene sequences were selected accordingly and aligned against the atemoya genome using TBtools (v2.483) [24]. To ensure specificity, we constructed a local atemoya database and conducted BLASTn searches using nucleotide sequences of reference genes from soursop, cherimoya [7], apple [8], cherry [9], populus [22], and tomato [23] as queries. Genes exhibiting ≥ 80% sequence similarity were retained for primer design. Six candidate reference genes—UBC, ACT7, ACT11, EF1α, TUB, and 18S—were selected for primer development. Primers were designed with the following parameters: primer length of 18–25 nucleotides, amplicon size ranging from 150 to 300 bp, melting temperature (Tm) between 55 and 60 °C, and GC content of 45–55%. The detailed characteristics of the primers used in this study are summarized in Table 1.

### 4.4. PCR

All primer pairs were validated via conventional PCR using 2× Magic Green Taq SuperMix (21502, Tolo Biotech Co., Ltd., Shanghai, China) following the manufacturer’s protocol. Amplification was carried out in a Mastercycler ×40 (Eppendorf AG, Hamburg, Germany) under the following thermal cycling conditions: initial denaturation at 95 °C for 3 min; 35 cycles of 95 °C for 15 s, 55 °C for 15 s, and 72 °C for 30 s; and a final extension at 72 °C for 5 min. The resulting amplicons were resolved by electrophoresis on 1.0% agarose gels and visualized using the ChemiDoc MP Imaging System (Bio-Rad, Hercules, CA, USA).

### 4.5. Standard Curve and qRT-PCR

Quantitative real-time PCR (qRT-PCR) was performed on a Quantagene q900MX system using Hieff UNICON^®^ Universal Blue qPCR SYBR Green Master Mix (11184ES03, YEASEN, Shanghai, China). Each 10-μL reaction mixture contained 5 μL of 2× Premix, 0.5 μL of cDNA template, 0.3 μL (10 μM) of each forward and reverse primer, and 3.9 μL of nuclease-free water. Standard curves were generated from 10-fold serial dilutions of cDNA, with each dilution tested in triplicate under each condition.

The amplification efficiency (E) of each primer pair was calculated using the formula: E = (10^(−1/slope)^ − 1) × 100 [16]. The resulting standard curve parameters are summarized in Table 1. All qRT-PCR reactions were performed in three technical replicates and three biological replicates, with each reaction containing 20 ng of cDNA per condition. The thermal cycling protocol consisted of an initial polymerase activation step at 95 °C for 10 min, followed by 40 cycles of denaturation at 95 °C for 15 s and annealing/extension at 55 °C for 60 s. Fluorescence signal acquisition was performed at the end of each extension step. Samples exhibiting a cycle threshold (Ct) value > 36 were excluded from further analysis. To confirm primer specificity, melting curve analysis was conducted over a temperature gradient from 55 °C to 95 °C at the conclusion of each run.

### 4.6. Transcript Stability Analysis

The Cq values were determined using a fixed threshold. The raw Cq values were then converted to relative quantities (Q) using the formula Q = E^−ΔCq^. Average stability (M) and pairwise variation (V) values were computed using the geNorm algorithm within the NormqPCR package (R environment) [10,12]. To comprehensively evaluate the stability of the candidate transcripts, we employed the RefFinder web tool (https://blooge.cn/RefFinder/) (accessed on 1 September 2026) [11], which integrates the algorithms of geNorm, NormFinder, BestKeeper, and the comparative ΔCt method to generate a comprehensive stability ranking based on the qRT-PCR data.

### 4.7. Normalization of AaPG and AaERF

To validate the stability rankings of the candidate reference genes, the expression profiles of two target genes, AaPG (a putative polygalacturonase homolog in tomato) and AaERF (an ethylene-responsive transcription factor homolog in banana), were examined. *A. atemoya* fruit were treated with ethephon for 12 h or 1-MCP for 4 days using the method described above, with untreated fruit at the corresponding time points serving as controls. Pulp tissues were collected at the end of each treatment, and the transcriptional levels of AaPG and AaERF were analyzed under the same experimental conditions. For relative quantification, the geometric mean of the three most stable reference genes and the three least stable genes (determined across all samples) were employed as normalization factors. Additionally, the optimal reference gene combination identified under all experimental conditions was also used for normalization. Relative expression levels were calculated using the standard E^−ΔΔCq^ method. All data were analyzed by Student’s *t*-test to compare each treatment group with its corresponding control, and results are presented as means ± standard deviation (SD).

## 5. Conclusions

Based on the comprehensive evaluation using all algorithms, *ACT11* and *ACT7* emerged as the most stable reference genes across different atemoya tissues, while *UBC* was preferentially ranked during fruit ripening and under various postharvest treatments. Collectively, our results indicate that *ACT7*, *UBC*, and *18S* are all suitable reference genes for atemoya gene expression normalization. Given the complexity of gene expression regulation across distinct tissues and the dynamic nature of climacteric fruit ripening, we recommend the combined use of *ACT7*, *UBC*, and *18S* as the preferred reference gene panel to ensure robust and reliable normalization for future RT-qPCR studies in atemoya, particularly those focused on tissue development and postharvest physiology.

## Figures and Tables

**Table 1:** List and description of the genes used in this study.

Gene Name	Gene ID	Accession Number	Sequence (5′–3′)	Amplicon Size (bp)	Primer Efficiency (%)	R^2^
*Ubiquitin carrier-like protein (UBC)*	AsquamosaB04T001274.1	PZ834014	F: GGAGGAAGAGAAGGAAGGAG	174	97.72	0.99
			R: AGATGACAGCGTTCCAGAG			
*Actin 11 (ACT11)*	AsquamosaB01T001612.1	PZ834015	F: TATTGTGAGCAACTGGGATGAC	202	92.03	0.99
			R: GTGAGAGAACAGCCTGGATAG			
*Elongation factor 1α (EF1α)*	AsquamosaB02T002408.1	PZ834016	F: CATTCAAGTATGCGTGGGTTCTTG	299	103.14	0.99
			R: CCATCTTGTTGCAGCAGCAAATC			
*18S ribosomal RNA (18S)*	AsquamosaB05T003203.1	PZ830889	F: CAACCATAAACGATGCCG	114	106.68	0.99
			R: TTCAGCCTTGCGACCATAC			
*Actin 7 (ACT7)*	AsquamosaB04T001488.1	PZ834017	F: CACTGGAATGGTCAAGGCTG	272	95.78	0.98
			R: AGCACTGGGTGTTCTTCAGG			
*Tubulin (TUB)*	AsquamosaB05T002062.1	PZ834018	F: ACGCTGCCAACAACTTTGC	199	90.61	0.99
			R: TTTCTTTCCATAGTCCACGGAG			

F and R means forward and reverse primers, respectively. R^2^ indicates the correlation coefficient.

**Table 2:** Summary of transcript stability values for the candidate reference genes, as evaluated by five distinct algorithms.

Approach	Gene Name	geNorm		Normfinde		ΔCt Method		BestKeeper		RefFinder	
		S	R	S	R	S	R	S	R	S	R
Tissue specificity	*UBC*	0.8610567	3	0.33	2	0.861	3	0.712	3	2.711	3
	*EF1α*	1.2773697	5	0.75	5	1.277	5	1.394	6	4.919	6
	*18S*	1.310983	6	0.94	6	1.311	6	0.319	1	3.834	5
	*ACT11*	0.7655851	1	0.23	1	0.766	1	0.831	4	1.414	1
	*ACT7*	0.8399177	2	0.40	3	0.840	2	0.990	5	2.340	2
	*TUB*	1.1417452	4	0.60	4	1.142	4	0.343	2	3.557	4
Maturation stages	*UBC*	1.324891	2	0.76	2	1.325	2	0.480	2	1.682	1
	*EF1α*	1.20703	1	0.58	1	1.207	1	0.807	3	1.732	2
	*18S*	1.351507	3	0.91	4	1.352	3	0.350	1	1.732	3
	*ACT11*	2.775855	6	1.9	6	2.776	6	2.069	6	6.000	6
	*ACT7*	1.475183	4	0.9	3	1.475	4	1.379	4	4.229	4
	*TUB*	1.625934	5	1.06	5	1.626	5	1.697	5	4.792	5
Cold storage	*UBC*	1.548246	1	0.83	2	1.548	1	0.607	2	1.861	1
	*EF1α*	1.660467	3	0.91	3	1.660	3	0.909	3	2.280	3
	*18S*	1.850972	4	1.23	5	1.851	4	0.511	1	2.115	2
	*ACT11*	2.095606	5	1.11	4	2.096	5	1.260	4	4.472	5
	*ACT7*	1.657675	2	0.61	1	1.658	2	1.556	5	2.515	4
	*TUB*	3.481759	6	2.2	6	3.482	6	3.180	6	6.000	6
Ethylene treatment	*UBC*	0.4735457	1	0.18	1	0.474	1	0.177	2	1.682	1
	*EF1α*	0.6752141	6	0.58	6	0.675	6	0.585	6	6.000	6
	*18S*	0.5886678	4	0.48	3	0.589	4	0.148	1	1.861	2
	*ACT11*	0.5863325	3	0.49	4	0.586	3	0.465	5	4.162	5
	*ACT7*	0.5095674	2	0.3	2	0.510	2	0.248	3	2.449	3
	*TUB*	0.6476802	5	0.51	5	0.648	5	0.345	4	3.162	4
1-MCP treatment	*UBC*	0.9447457	1	0.62	2	0.945	1	0.349	3	1.565	1
	*EF1α*	0.9457932	2	0.3	1	0.946	2	0.787	4	2.378	3
	*18S*	1.0346461	3	0.82	4	1.035	3	0.317	2	2.060	2
	*ACT11*	1.1851452	5	0.84	5	1.185	5	0.179	1	2.943	4
	*ACT7*	1.1830308	4	0.78	3	1.183	4	1.451	5	4.472	5
	*TUB*	1.6902674	6	1.44	6	1.690	6	1.991	6	6.000	6
Global analysis	*UBC*	1.850394	1	0.62	1	1.850	1	0.476	4	2.000	2
	*EF1α*	3.125469	5	1.2	4	3.125	5	2.030	5	5.000	5
	*18S*	2.106917	4	1.22	5	2.107	4	0.154	1	2.000	3
	*ACT11*	3.946634	6	2.32	6	3.947	6	3.136	6	6.000	6
	*ACT7*	1.860116	2	0.84	3	1.860	2	0.333	2	1.682	1
	*TUB*	2.030089	3	0.77	2	2.030	3	0.447	3	3.000	4

geNorm (average expression stability M), NormFinder (stability value), the ΔCt method (mean standard deviation), BestKeeper (standard deviation of crossing point values, SD [±CP]), and RefFinder (comprehensive ranking). For each algorithm, “S” denotes the stability value and “R” denotes the corresponding rank.

## Data Availability

The original contributions presented in this study are included in the article/Supplementary Materials. Further inquiries can be directed to the corresponding author.

## Supplementary Materials

The following supporting information can be downloaded at: https://www.mdpi.com/article/10.3390/ijms27188114/s1.

## Abbreviations

The following abbreviations are used in this manuscript:

1-MCP	1-methylcyclopropene
PG	Polygalacturonase
